# Source apportionment of ambient pollution levels in Guayaquil, Ecuador

**DOI:** 10.1016/j.heliyon.2024.e31613

**Published:** 2024-05-21

**Authors:** Mario Patiño-Aroca, Tomás Hernández-Paredes, Carlos Panchana-López, Rafael Borge

**Affiliations:** a Escuela Superior Politécnica del Litoral, ESPOL, Campus Gustavo Galindo, Km 30.5 Vía Perimetral, Guayaquil, 090902, Ecuador; b Department of Chemical & Environmental Engineering, Universidad Politécnica de Madrid (UPM), C/ José Gutiérrez Abascal 2, 28006 Madrid, Spain; c Universidad Agraria del Ecuador, Facultad de Ciencias Agrarias “Dr. Jacobo Bucaram Ortiz”, Av. 25 de Julio y Pío Jaramillo, P.O. Box 09-04-100, Guayaquil, Ecuador

**Keywords:** Urban air pollution, Emission inventory, Air quality modeling, WRF/CALMET/CALPUFF modeling system, Source contribution

## Abstract

In this study, the relative contributions of main emission sources to the typical ambient concentrations of key pollutants, such as sulfur dioxide (SO_2_), nitrogen dioxide (NO_2_), carbon monoxide (CO), and particulate matter (PM_10_ and PM_2.5_) in Guayaquil, Ecuador, were investigated. A previous urban emissions inventory for mobile sources was expanded to include other transportation means and main industrial activities using the EMEP/EEA methodology to achieve this objective. The WRF/CALMET/CALPUFF modeling system was used to simulate the annual spatiotemporal distribution of air pollution in the city. According to the model, NO_2_ concentrations exceed the yearly value and 1-h Ecuadorian standards (40 and 200 μg/m^3^) in 1 % and 6 % of the cells of the modeling domain, respectively. These hotspots related to local sources were located in the northwest center of the city. The contributions of the manufacturing sector, thermal power plants, ports, airports, and road traffic were assessed individually, and the results indicated that air quality in the study area was strongly dominated by road traffic. The contributions of NO_2_, CO, PM_10_, and PM_2.5_ at the city level reached 76 %, 96 %, 90 %, and 92 % of the annual mean, respectively. In the case of SO_2_, the manufacturing sector made the most significant contribution (75 %), followed by thermal power plants (16 %). Furthermore, an analysis at 14 specific locations across Guayaquil identified spatial variations that may support the design and development of an air quality monitoring network for the city.

## Highlights

• Emissions estimated for mobile sources, power plants, and industry in Guayaquil.
• Road traffic is the most important emission source in the city.
• Pollution dispersion modeled with the WRF/CALMET/CALPUFF over 1-yr period.
• Road traffic responsible of 92 % of PM_2.5_ and 76 % of NO_2_ ambient concentration.
• 91 % of SO_2_ concentration attributed to manufacturing sector and power plants.

## 1 Introduction

Previous studies in various continents have shown that emission rates are closely related to economic growth, with an inverse or U-shaped relationship between income levels and environmental degradation [1]. Therefore, governments must develop sustainable environmental policies, in addition to boosting economic growth. This involves minimizing emissions from thermoelectric generation, manufacturing, and the road and off-road transport sectors (both passengers and goods) [[2], [3], [4], [5]], as they are directly linked to air quality and exposure in urban areas and strongly impact morbidity and mortality in societies [6], subject to particular conditions [7]. As a result, the World Health Organization has developed air quality guidelines for pollutants such as CO, NO_2_, SO_2_, O_3_, PM_2.5_ and PM_10_ [8], which have key implications for public health because of their association with cardiovascular and respiratory diseases and other health outcomes, including premature mortality [9,10]. Therefore, they can be used as standard references in national and local regulations.

Air quality monitoring networks are key to assessing whether concentrations exceed healthy levels in urban areas [11], but they may entail a high initial investments and considerable operational costs [12] and should be carefully considered. Similar to many other cities in developing economies, there are no permanent air quality monitoring stations for assessing pollution levels. A recent study in Guayaquil monitored PM_10_ and PM_2.5_ in Guayaquil from September 1, 2015, to November 17, 2016, using a particle counter [13]. The authors found that the average concentrations of both pollutants remained below dangerous levels, although continuous monitoring throughout the study period revealed that the maximum values often exceeded the permissible limits set by Ecuadorian Regulations.

Air quality models are widely used to obtain pollutant concentration maps to support decision-making, including the design and assessment of air monitoring strategies [[14], [15], [16], [17], [18], [19]]. Such approaches based on modeling have lower cost compared to monitoring networks, in addition to the advantage that modeling tools have of predicting the effect of any intervention or changes in activity patterns in a city. Although emission inventories can provide information on emissions, source apportionment methods, such as brute force or direct decoupling, among others [[20], [21], [22]] are needed to understand the relationship between emission sources and air quality. The fundamental difference between these methods lies in the manner in which the interactions of some sources with others are considered. Often, these relationships are non-linear, especially for secondary pollutants such as O_3_ or a large fraction of PM_2.5_ [21], making the attribution of ambient concentration levels highly complex. The problem is considerably simplified when the modeled species are assumed to be passive and, therefore, there are no chemical reactions. Under this hypothesis, apportionment becomes unambiguous and perfectly additive [20]; therefore, a separate analysis for each source can be performed and additivity is guaranteed. Although this is conceptually much simpler and the relationship between emission sources and ambient pollution levels is robust, it introduces considerable uncertainties because the concentration of secondary species remains unexplained. Of note, any source apportionment method, regardless of the underlying assumptions, should be supported by a consistently complete and detailed emission inventory [[22], [23], [24]], This may prove challenging for developing countries owing to the lack of detailed local data on emission factors and economic activity [24]. New alternative methodologies for estimating atmospheric air pollution, such as satellite images for remote sensing, including Landsat ETM+ [25] and TROPOspheric Monitoring Instrument, which can detect vertical emission columns [26], may support the estimation of emissions. However, these techniques are still in the development stage and are beyond the scope of this research.

The lack of emissions may be a major reason for the scarcity of source apportionment studies in South America. While the application of attribution methods based on state-of-the-art chemical transport models is common in the US and Europe [21], the few previous studies that have analyzed the origin of ambient pollution levels in this region are based either on receptor models, such as the Positive Matrix Factor method [27,28], or statistical approaches, such as Principal Component Analysis [29]. All these studies relied on ad-hoc measurements and were limited to PM or some of its components.

In this study, an emission inventory was developed for the city of Guayaquil, Ecuador, considering the main emission activities according to previous studies [30]. The inventory of mobile sources compiled by Patiño-Aroca et al. (2022) [31] was the starting point of this study. Our research group quantified and spatially allocated emissions from road traffic in the city by using an International Vehicle Emissions (IVE) model. This study's results suggested that higher emission rates occur in densely populated areas of the city and are mainly linked to buses and private vehicles. High specific emission rates were observed for all vehicle segments owing to relaxed emission standards (Euro II or less). In this study, the emission inventory for mobile sources was complemented by an updated estimate of the emission rates of air pollutants emitted by thermal power plants, manufacturing, ports, airports, and transportation sectors, for 2018. A modeling system was used to simulate the spatiotemporal variations in ambient concentrations and to analyze each source's contribution to ambient concentration levels, presenting the first explanation of the origin of air pollution levels in the city. This study focused on the most abundant pollutants typically related to anthropogenic activities and economic growth [32], which have a significant impact on human health [33]. SO_2_, NO_2_, CO, PM_10_, and PM_2.5_ were considered primary/passive pollutants as a first step toward understanding the source apportionment of ambient air quality in this urban area.

To the best of our knowledge, this is the first apportionment analysis in Ecuador. In addition to generating new information regarding air pollution in Guayaquil, the results of this study specifically intend to support the design of an air quality monitoring network, which is currently nonexistent. This would be instrumental in the development of a comprehensive air quality management framework that will ultimately contribute to assessing the exposure levels and the effects of future emission abatement measures in the city. Meanwhile, the methodology developed in this study can be used in other cities worldwide with a similar degree of development in air quality management. Specifically, we present a source apportionment methodology that may be a reasonable compromise between accuracy and relatively low input information requirements, which may be important for many other urban areas where data scarcity poses an additional challenge in creating a link between emission sources and ambient pollution levels.

## 2 Methodology

**Fig. 1:**
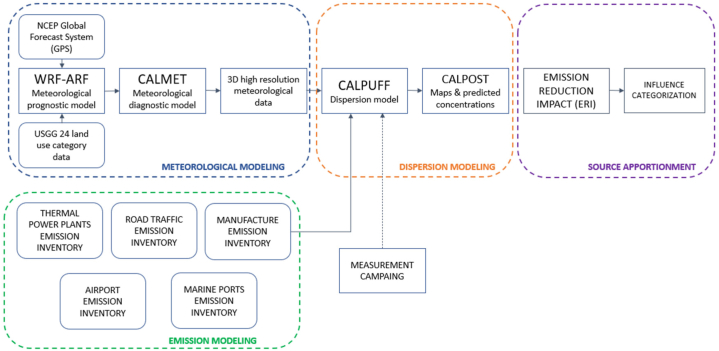
Methodological framework.

As summarized in Fig. 1, the methodology used in this study consisted of four main components: i) the development of an emission inventory for stationary and mobile sources of SO_2_, NOx, CO, PM_10_, and PM_2.5_; ii) the generation and evaluation of high-resolution meteorological information for the study area; iii) the application of an air quality modeling system; and iv) the assessment of the contribution of each emission sector to the annual concentration. In addition, two experimental campaigns were conducted to obtain a minimum understanding of the current ambient concentrations in the city.

### 2.1 Study area

**Fig. 2:**
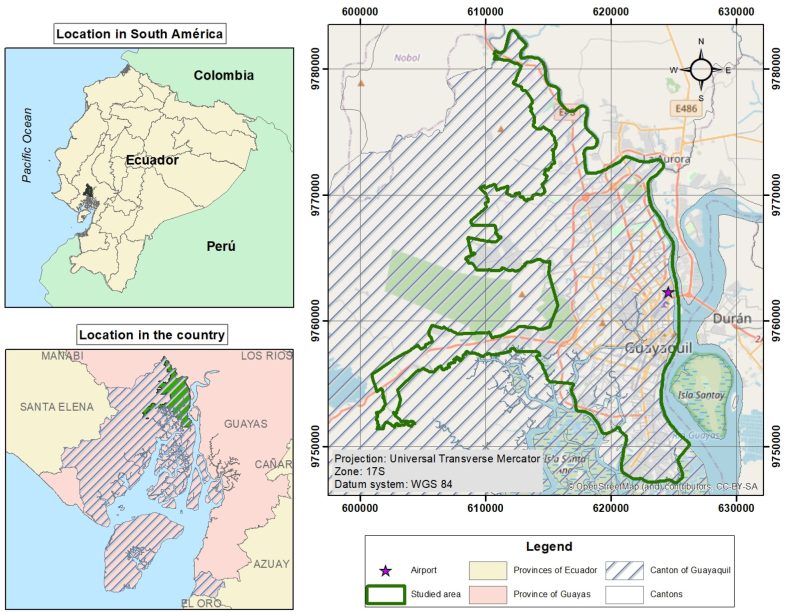
Urban area of Guayaquil.

Guayaquil was selected as the study area because of its relevance and is the second most populated city in Ecuador (after Quito, the capital of the country), with an approximate population of 2,700,000 inhabitants, according to population figures projections to 2020 from the National Institute of Statistics and Censuses (INEC). It is the main maritime port of Ecuador, with a mean elevation of 4 m above sea level and geographic coordinates of 2°11′24″ S, 79°53′15″ W (Fig. 2). It has an urban territorial extension of 344.5 km^2^ and a mean population density of approximately 7900 inhabitants/km^2^. Industrial activity in the automotive sector has undergone notable changes over the last five years, with an average increase of 10 % in the vehicle fleet [31].

The climate is defined by clear seasonality during two periods. The mean annual temperature is 25 °C, with a maximum monthly temperature of 35 °C (March) and a minimum monthly temperature of 20 °C (August). The mean annual rainfall in the last ten years reached 1200 mm, which was concentrated during the rainy season from January to May. Meanwhile, an annual average of 75 % relative humidity and a wind speed of 3.5 m/s were reached, with 4 m/s in the dry season (June to December) and 2.9 m/s in the rainy season (January to May), and the predominant wind direction is from the southwest.

### 2.2 Emission inventory

**Fig. 3:**
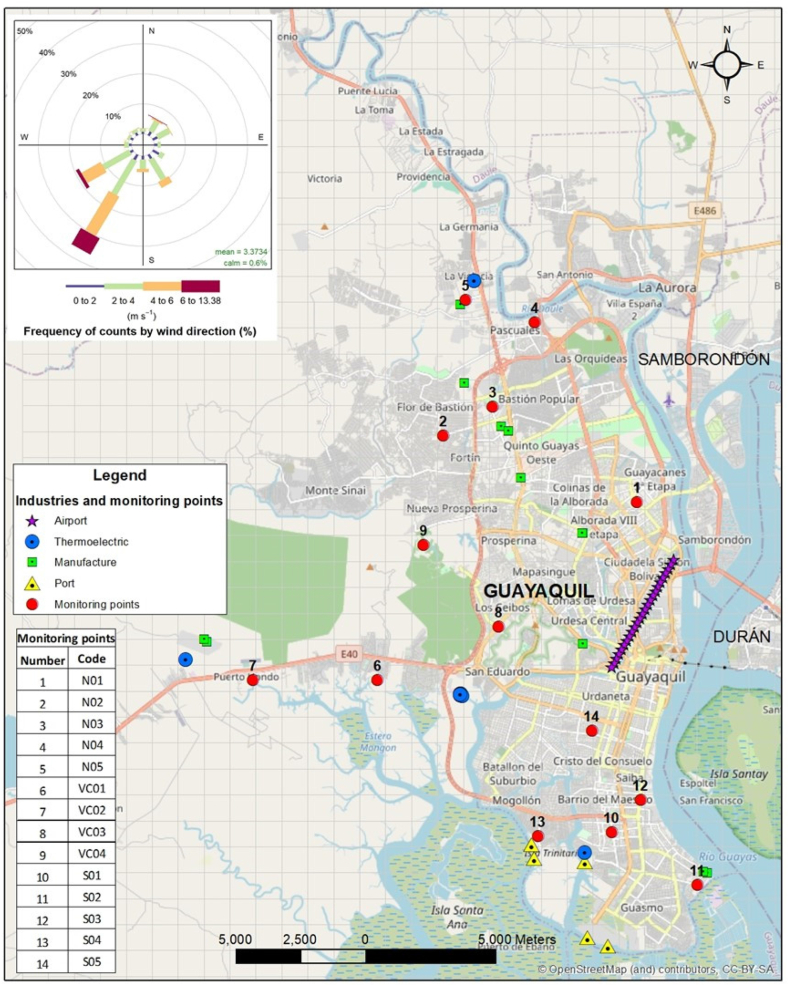
Location of the point sources and experimental campaign monitoring sites. The wind rose for the year 2018 according to the observations at “José Joaquín de Olmedo” International Airport is shown as well.

The emissions inventory was the main component and first step of this methodological framework; therefore, careful analysis of the main mobile and stationary sources was necessary to obtain a reasonable initial estimation. The most important emission sources, identified within the limits of the urban area, were classified according to their origin as follows (excluding road traffic): industry (production of processed foods, beverages, paper, rubber, plastic, non-metallic minerals, and metallic products and their derivatives), thermoelectric power plants, seaports, airports, and exploitation in the mining sector, although mining was not considered in this study because of the lack of detailed information. Fig. 3 shows the locations of each of these stationary sources along the city's road network. In total, 15 manufacturing plants, 14 thermal power plants and five ports were considered point sources for modeling purposes in the local inventory. Emissions from the airport and transport sectors were considered as the area sources for modeling purposes.

#### 2.2.1 Stationary sources

For all stationary sources, the methodological approach published in the 2019 Air Pollutant Emission Inventory Guidebook [34] of the European Environment Agency (EEA) was followed.

Emissions from the manufacturing sector were estimated by separating them into two types: combustion and material transformation. Table S1 in the supplementary material shows the manufacturing industries according to the International Standard Industrial Classification System, version 4 [35], which operated in 2018 within the city of Guayaquil. Tables S2, S3, and S4 show the emission factors applied, and Table S5 shows the properties of the fuel used in the industry. For SO_2_ combustion emissions, the mass balance method was used. Activity rates, fuel consumption, manufactured products, and raw materials used in the manufacturing industry were obtained from the Business Structural Survey of the INEC of Ecuador [36].

The emission sources and emission factors for the power generation sector are shown in Tables S6 and S7 of the supplementary material, respectively. SO_2_ emissions were determined using the same method used for the manufacturing sector. Fuel consumption was obtained from the Annual and Multi-annual Statistical Report of the Ecuadorian Electric Sector of the Electricity Regulation and Control Agency [37].

The Tier 3 approach was used for the emission inventory of aeronautical operations. Emissions in this sector relate to the activity carried out at the José Joaquín de Olmedo international airport located in Guayaquil for both domestic and international flights, considering the steps of Landing Take Off (LTO), Taxi in, and Taxi Out, where information provided by the airport operators for the year 2017 was obtained, the same which was extrapolated to the year 2018 and accepted as a reasonable approximation with the help of expert judgment. Additional details are provided in the supplementary material.

Regarding the inventory of emissions in ports, Tier 3 was followed. Our inventory only considered the hoteling and maneuvering stages developed within the study domain. Table S8 of the supplementary material, lists the emission factors for this sector.

#### 2.2.2 On-road emission approach

An inventory of road vehicle emissions with a base year of 2018 was estimated for Guayaquil [31] using the IVE model. Of note, the estimate used in this study corresponded to exhaust emissions, that is PM emissions from brake, and tire wear or resuspension processes were not included. Additional information and aggregate emission results for each vehicle type can be found in the supplementary material (Table S10). The reader is referred to Ref. [31] for further details on the methodology.

### 2.3 Air quality modeling system and model setup

#### 2.3.1 Modeling system

In this study, the WRF/CALMET/CALPUFF modeling system was used to investigate the spatiotemporal distribution and contribution of five different source categories to the concentrations of SO_2_, NO_2_, CO, PM_10_, and PM_2.5_ in Guayaquil.

Other authors [38,39] have performed hybrid simulations to obtain a better performance in describing meteorological variables using CALMET by feeding the model with surface and upper-air observations, in addition to obtaining results from forecast models such as the WRF [40].

However, only information from a surface meteorological station in the study area was available for the base year corresponding to that of José Joaquín de Olmedo Airport, and the only upper-air station located in the city operated once a day, three days a week. Therefore, in this study, to generate the required spatial and temporal resolutions, the outputs obtained from the state-of-the-art mesoscale model WRF (WRF-ARW 3.7.1) [41].

An advantage of using this system is that the meteorology in the WRF format in combination with the CALMET diagnostic meteorological module [42] allows for the refinement of meteorological fields considering local topography and land use; therefore, local atmospheric phenomena and key variables, such as wind fields or mixing height [43], could be reproduced with the necessary precision in the study area.

The data in the WRF output files were converted into a format compatible with CALMET (version 5.8.5) (www.src.com) using the CALWRF interface program (version 2.0.1). The CALWRF output formed a grid covering the modeling domain with a resolution of 4 km.

The meteorological fields obtained from the WRF/CALMET were used to run the CALPUFF air quality model [44] used in this study to investigate the spatiotemporal distribution of the main pollutants in Guayaquil. CALPUFF is a multi-layered, multi-species, and unsteady-state Lagrangian puff model that simulates the effects of time- and space-varying meteorological conditions during pollutant transport, dispersion, transformation, and removal. The EPA-approved version of CALPUFF (version 5.8.5) (www.src.com) included in the CALPUFF View (version 9.0.1)® interface was used in this study.

#### 2.3.2 Model setup

The nested domains used for the WRF (Fig. S1) and the vertical and horizontal structures are summarized in Tables S11 and S12 of the supplementary material. Table S13 of the supplementary material lists the configuration and parameters used to apply the WRF model.

CALMET was configured with a 50 km × 50 km modeling domain centered at the location of the José Joaquín de Olmedo International Airport (2.15° S, 79.88° W) and covered the entire city of Guayaquil, with a horizontal resolution of 1 km × 1 km and ten layers with 20 m, 40 m, 80 m, 160 m, 320 m, 640 m, 1200 m, 2000 m and 3000 m. A temporal resolution of 1 h was used to describe the temperature, wind direction, mixing height, and precipitation.

**Fig. 4:**
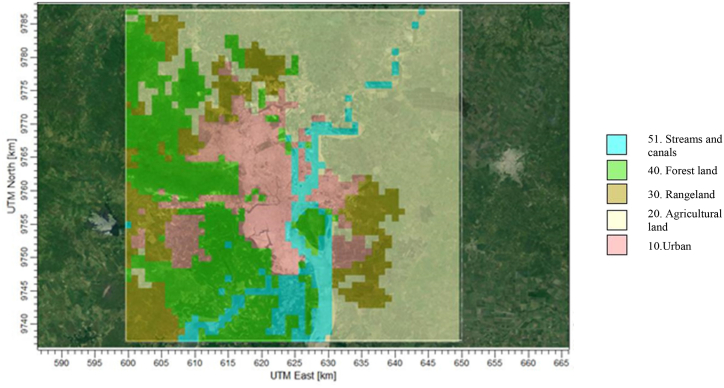
Updated land use data from geoportal MAGAP – Ecuador (year 2013).

Land elevation maps were obtained from the Shuttle Radar Topography Mission SRTM1 (Global 30 m), version 3, and SRTM30 (Global 900 m) [45], and land use data was obtained from the Global Land Cover Characterization (South America 1 km) [46]. Considering that the information available through satellite images of land use for Guayaquil corresponded to the year 2000, it was updated based on the information available on the geoportal of the Ministry of Agriculture, Livestock, Aquaculture, and Fisheries of Ecuador corresponding to the year 2013 (Fig. 4).

The CALPUFF modeling domain was identical to that of the previously discussed CALMET, and all simulations were performed for an annual period corresponding to the base year 2018 with a temporal resolution of 1 h.

This study selected the “puffs” method to represent pollutant emissions. The calculation of turbulent transport relied on the Pasquill–Gifford–Turner coefficient method for rural areas and the McElroy-Pooler coefficient for urban areas [47,48], which is the default option. No chemical reactions or deposition were considered.

#### 2.3.3 Emission adaptation for modeling

Considering that high spatial and temporal resolutions are required for air quality modeling applications [49,50], all emissions were spatially and temporally allocated over a 50 km × 50 km modeling domain with a spatial resolution of 1 × 1 km and a temporal resolution of 1 h as an input to CALPUFF.

Regarding the spatial distribution, all point sources were georeferenced within the modeling domain and associated with the parameters of the height and diameter of the stack, emission rate, velocity, and gas outlet temperature. For the airport sector, these were distributed in 25 area sources along the length of the runway, at the beginning at ground level, and then staggered until reaching a height of 300 m, according to the methodology proposed in Ref. [51]. Finally, for the road transport sector, the result of the spatial distribution of emissions in a modeling domain of 50 km × 50 km with a resolution of 1 km^2^ from the total emissions of each pollutant was used, as shown in Ref. [31].

The temporal variability of point and area sources was considered, except for sources in the manufacturing sector. Owing to a lack of information, a constant temporal profile was considered for these industrial facilities. For the case of thermoelectric power plants, information on fuel consumption and monthly gross production was assessed to determine the monthly coefficient of variation. For ports, the coefficient of monthly variation was determined from information on the number of monthly arrivals and vessel departures. For aircraft emissions, the monthly emission coefficient of change was determined from the monthly LTO cycle data for each aircraft model (Table S9). Finally, the daily variation coefficient for the road transport sector was determined based on the results obtained from the emissions inventory presented in Ref. [31].

The supplementary material provides details of the emission rates and other relevant modeling parameters for the manufacturing point sources (Tables S14 and S15), power plants (Table S16), and ship emissions, which were used as point sources (Table S17), and the airport, which was considered as a group of area sources (Table S18).

Considering the complexity and density of the road network, the representation of emissions from mobile sources in CALPUFF required special treatment, as the input data for this type of line-area source are limited to straight lines, and the maximum number of roads is limited to 24 [52]. In a study conducted by Patiño-Aroca [31], considering the results obtained from the total annual emissions, the spatial distribution, length and type of roads, and intensity of vehicular traffic by road type were used as surrogate data. To calculate the emission allocation factors for each grid cell, they weighted the intersected road length according to the road type (highways, avenues, and streets), thus enhancing the primary roads, highways, and avenues, and, to a lesser extent, the secondary roads, that is, the streets. Finally, for air quality simulation, only cells that generated emissions were considered, resulting in 348 area sources.

The maximum number of area sources allowed by CALPUFF is 200. In this study, we relied on the spatiotemporal distribution of vehicular emissions from Ref. [31], which resulted in 348 area sources; therefore, for this study, the number of sources was divided into two equal parts, and then the sum of the two simulations were summed using the CALSUM tool provided by the model for such cases.

The Ambient Ratio Method (ARM) [53] was used to transform the simulated NOx concentration into NO_2_. In the absence of NO_2_ and NOx monitoring data, an NO_2_/NOx ratio of 0.75, in accordance with the EPA's guidelines, was assumed [54]. This conversion was applied during the post-processing stage using CALPOST (version 6.221) (www.src.com). This method is a simple method for estimating the net equilibrium of complex photochemical processes and estimate NO_2_, largely a secondary species. Although it may be more accurate than the approach followed for PM, where the secondary fraction [55] is completely neglected, it may introduce uncertainties in the NO_2_ estimates. Considering the lack of monitoring data and that the primary goal of this study was to estimate the contribution of local sources, background concentrations were not considered. The simulation results represent air quality levels originating only from emissions originating in the urban area of the city.

Notably, post-processing in this study was configured to obtain annual concentrations. Although source apportionment may change depending on the averaging period, the annual mean is deemed a suitable first approximation because it reflects the aggregated contribution of each source across the year and is the most relevant averaging period for long-term health effects.

Regarding the definition of the receptors, in addition to the receptors located in the center of each grid cell (2500 receptors), 14 discrete receptors were defined (see Table S19 of the supplementary material) corresponding to the points where a measurement campaign was carried out.

Finally, five simulations were performed, one for each source type: thermal power plants, ports, airports, and road transport. In addition, using the CALSUM tool, the spatiotemporal distribution maps of the total concentration could be determined owing to the combined operation of the five sources by direct addition.

### 2.4 Experimental field campaigns

Given the absence of observed air quality data for Guayaquil, it was impossible to evaluate the dispersion model. To mitigate this limitation and consider the relatively low inter-annual meteorological variations in the study area, experimental field campaigns were conducted in October 2020 and March 2021 to obtain a rough idea of the ability of CALPUFF to reproduce the order of magnitude of the concentrations in the study area. Notably, these dates were not affected by mobility imposed on the city to prevent the spread of COVID-19. To address this task, a diffusive passive radial collector, Radiello [56], was used to evaluate the concentrations of NO_2_ and SO_2_ over a time interval of seven days, in addition to use of a low-flow gravimetric sampler, BGI PQ200, in a 24-h measure campaign combined with measurements performed by Dylos 1700, considering its weakness as a laser-based measurement method [57].

Monitoring points were selected based on a previous measurement campaign carried out in 2012 in Guayaquil [58], which considered 10 points throughout the city. In this study, four additional sampling points were added (Fig. 3), three of which considered the population density of the city and the fourth was considered as a background location, which also allowed for determining how air quality levels in the city have evolved. Notably, selecting these specific locations may compromise the spatial representativeness of the measurements, but it guarantees temporal comparability and, thus, allowed for the assessment of pollution trends over the last decade.

### 2.5 Source apportionment

The modeling system described in Section 2.3.1 has been used in several source contribution studies [17,52,[59], [60], [61], [62]]. Because of the hypothesis regarding chemical reactions, the interactions among species/sectors could be neglected, and the contribution of each source to the concentration of the set of all sources at any receptor was determined individually [17,63]. Notably, this may be questionable only for NO_2_ because we considered the oxidation of NO using a simple parameterization [53]. Unlike other methods commonly used in dispersion models [64], the ARM does not explicitly consider the O_3_ level, which controls NO titration or the production of secondary NO_2_. This implies that we neglected a factor that may be critical in explaining the oxidation capacity of the atmosphere in urban areas [65], but the additivity assumption was not compromised, and the uncertainty was limited to that of the ARM itself.

The relative contribution (dimensionless) to the concentration of source i for pollutant j for a given period k (in this study, the annual mean) at receptor n was characterized as S (i, j, k, n) and was calculated according to Eq. (1):

(1) $$S_{\left(i,j,k,n\right)}=\frac{C_{\left(i,j,k,n\right)}}{\sum_{i=1}^{5}C_{\left(i,j,k,n\right)}}$$

where: i = source (1–5), j = pollutant (1–5), k = period (annual), and n = receptor (1–14).

The source apportionment was assessed using 14 discrete receptors defined in CALPUFF that coincided with the monitoring points in the experimental campaign (Fig. 3). An estimation of the distribution of sources was also made considering the annual concentration for the entire city, that is, by averaging the results in the 2500 grid cells in the modeling domain.

## 3 Results and discussion

### 3.1 Emissions inventory

Table 1 summarizes the sectoral emissions of the main air pollutants in Guayaquil for the base year, 2018.

**Table 1:** Emission inventory of air pollutant for Guayaquil (year 2018).

Sector	SO_2_		NO_x_		CO		PM_10_		PM_2.5_	
	t/year	%	t/year	%	t/year	%	t/year	%	t/year	%
Thermal Power Plant	9310	71.3	2370	4.4	193	0.1 %	319	3.8 %	245	3.0 %
Manufacturing Industry	2821	21.6	3374	6.3	3342	1.4 %	190	2.3 %	123	1.5 %
Road traffic	698	5.3	46,397	86.6 %	237,147	98.3 %	7679	92.3 %	7679	93.9 %
Airport	24	0.2 %	320	0.6 %	511	0.2 %	3	<0.1 %	3	<01 %
Ports	202	1.5 %	1130	2.1 %	176	0.1 %	125	1.5 %	125	1.5 %
Total	13,055	100 %	53,591	100 %	241,369	100 %	8316	100 %	8175	100 %

**Fig. 5:**
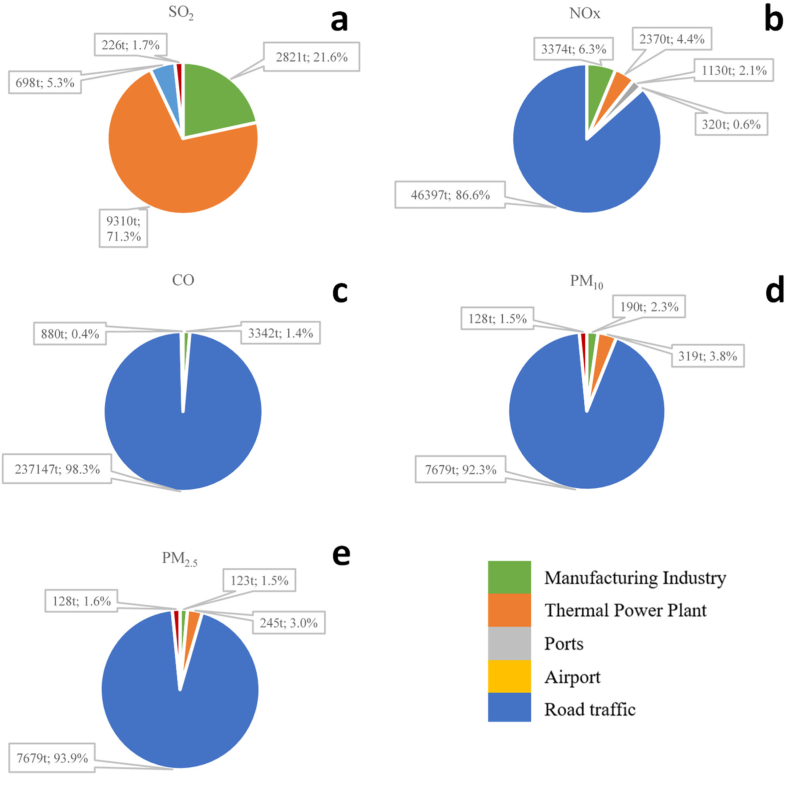
Sectoral contributions to air pollutant emission in Guayaquil for the base year of 2018 for the main pollutants: SO_2_ (a), NO_X_ (b), CO (c), PM_10_ (d), and PM_2.5_ (e).

Thermoelectric plants were responsible for 71.3 % of SO_2_ emissions (9310 t). The second-highest emission share for this pollutant was that of the manufacturing sector, with 21.6 % of the emissions (2821 t), followed by road traffic with 5.3 % (698 t) (Fig. 5).

Road traffic was the main emission source for the remaining pollutants, with contributions of 86.6 % (46,397 t), 98.3 % (237,147 t), 92.3 % (7679 t), and 93.9 % (7679 t) for NOx, CO, PM_10_, and PM_2.5_, respectively. For NOx, it was followed by the manufacturing sector at 6.3 % (3374 t) and thermal power plants at 4.4 % (2370 t) in third place. For CO, it was also followed by the manufacturing sector, with 1.4 % of the emissions (3342 t), and for PM_10_ and PM_2.5_, it was followed by thermal power plants at 3.8 % (319 t) and 3.0 % (245 t), respectively, while the manufacturing industry contributed 2.3 % (190 t) and 1.5 % (123 t), respectively. Ports and airports contributed approximately 2 % or less to the total emissions of all pollutants.

These results clearly indicated that mobile sources were the largest emitting sector in 2018 in Guayaquil, surpassing thermoelectric plants and the manufacturing sector combined by 20 times for pollutants such as PM_2.5_, even though our inventory only considered exhaust emissions. The opposite situation was observed regarding SO_2_ emissions, where thermoelectric plants and the manufacturing sector together contributed 93 % of the emissions, compared to the 5.3 % from mobile sources. This was mainly because the fuels used for producing electrical energy and in the industry have a high sulfur content.

Emissions from the mining sector are presumably an essential source in terms of the emission of particulate matter, mainly PM_10_ [[66], [67], [68]]. However, at the time of this study, insufficient information was available to include them in the 2018 emissions inventory.

### 3.2 Meteorological simulations

**Fig. 6:**
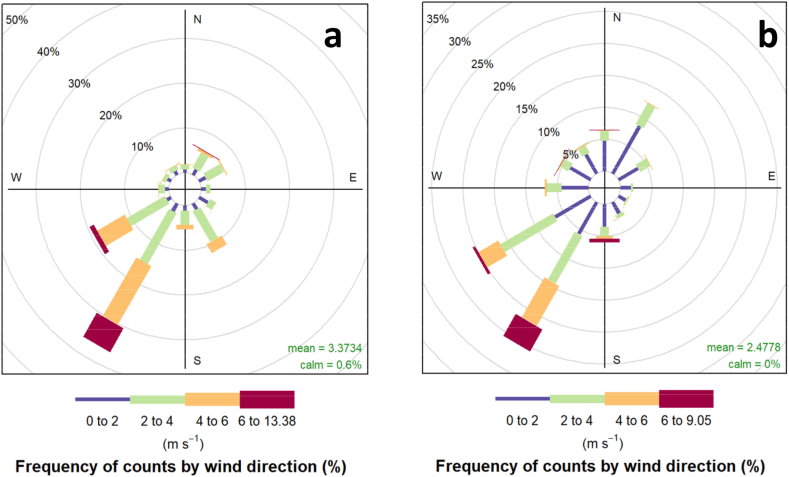
Observed (a) and modeled (b) by WRF/CALMET multianual (2015–2018) wind roses.

The performance of the WRF/CALMET meteorological model was evaluated by comparing simulated and observed values at a meteorological station located at the city airport. Fig. 6 shows that the multi-annual wind occurred between 2015 and 2018. The predominant wind direction was from the southwest, and in the rainy season, another important wind direction existed from the northeast. In general, the results for the direction (Fig. 6a) and speed (Fig. 6b) of the wind showed good agreement between the observed and simulated data. The same results stratified by year and for the two seasons are shown in Fig. S2 in the supplementary material.

Four statistical indices were selected to provide a quantitative assessment of the temperature and wind speed simulations: the mean bias (MB), mean absolute error, root mean square error, and index of agreement (IOA).

Table 2 shows the results of these statistics for 2018 and a comparison with the reference benchmarks proposed by Emery [69] (originally for 24-h mean values).

**Table 2:** Statistics for temperature and wind speed evaluation for 2018.

Temperature @ 2 m	MAE [K]	BIAS [K]	RMSE [K]	IOA
Reference standards	≤2	≤±0.5		≥0.8
**Annual**	**1.44**	**0.34**	**0.02**	**0.90**
Rainy season	1.40	−0.70	0.03	0.90
Dry season	1.47	1.07	0.03	0.90
**Wind speed @ 10 m**	**MAE [m/s]**	**BIAS [m/s]**	**RMSE [m/s]**	**IOA**
Reference standards		≤±0.5	≤2	≥0.6
**Annual**	**1.70**	**−0.97**	**2.07**	**0.60**
Rainy season	1.40	−0.79	1.74	0.70
Dry season	1.47	−1.09	2.27	0.54

The temperature data showed good correspondence according to this benchmark, although the largest deviation in wind speed was observed. Overall, temperature was overestimated with an annual mean value of 0.34 K and wind speed was underestimated, with a yearly MB value of −0.97 m/s. The annual IOA values were 0.90 for temperature and 0.60 for wind speed, indicating a comparatively poor model performance for wind but a good correspondence to the benchmarks. This was consistently observed when the analysis was extended to a four-year period (Fig. S2 of the supplementary material). Finally, it could be concluded that the behavior of the adjustment statistics for the meteorological variables was generally better in the rainy season than in the dry season because higher wind speeds were slightly underestimated in the latter.

### 3.3 Air quality simulation

The annual concentration was estimated along with the mean concentrations for the dry and rainy seasons to understand the dispersion of pollutants and spatial gradients in the modeling domain.

**Fig. 7:**
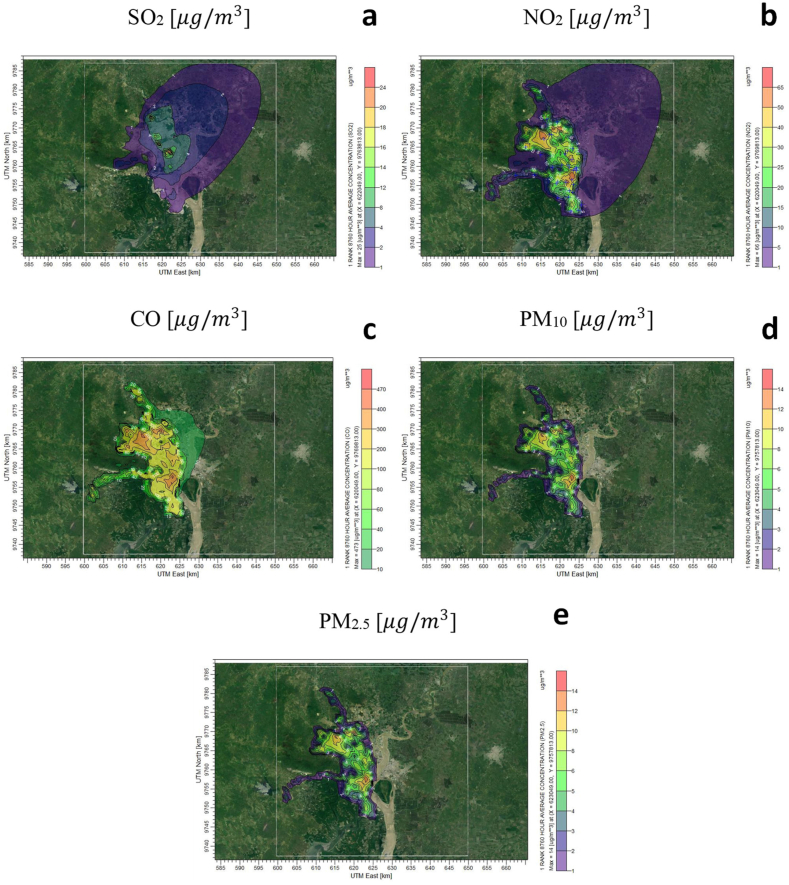
Spatial distribution of the simulated annual mean concentration fields for the main pollutants: SO_2_ (a), NO_2_ (b), CO (c), PM_10_ (d), and PM_2.5_ (e).

Considering the wind patterns described in the previous section, the pollutants were mainly dispersed to the northeast, although the maximum hourly mean (not shown) did not exhibit such a consistent pattern. Fig. 7 shows iso-concentration maps of the pollutants used in this study.

According to the model, the NO_2_ concentrations (Fig. 7b) exceed the value of the annual Ecuadorian standard (40 μg/m^3^) by 1 % of the cells of the modeling domain located in the northwest and the city downtown and by 6 % of the 1-h standard (200 μg/m^3^). Finally, in the case of SO_2_ (Fig. 7a), the map showed a different dispersion pattern, given that the primary emission sources were stationary point sources, unlike the other pollutants, which were dominated by the transport sector.

In general, owing to the homogeneity of wind fields throughout the year, the dispersion patterns of primary pollutants were very similar regardless of the time of year (Fig. S3). In addition, according to CALMET, the mean mixing heights were very similar in the dry and rainy seasons (400 m and 320 m, respectively).

Unlike the meteorological simulation, the air quality model performance could not be assessed owing to a lack of observational data. However, the information generated during the experimental campaign may provide a reference for the current concentrations in the city. There was only one measurement of PM_10_ and PM_2.5_ at each of the 14 monitoring points, corresponding to a one-to-three-day period. This prevented adequate quantitative statistical evaluation. Nonetheless, a qualitative assessment of the measured and observed data is presented below, even though neither dataset is strictly comparable.

**Fig. 8:**
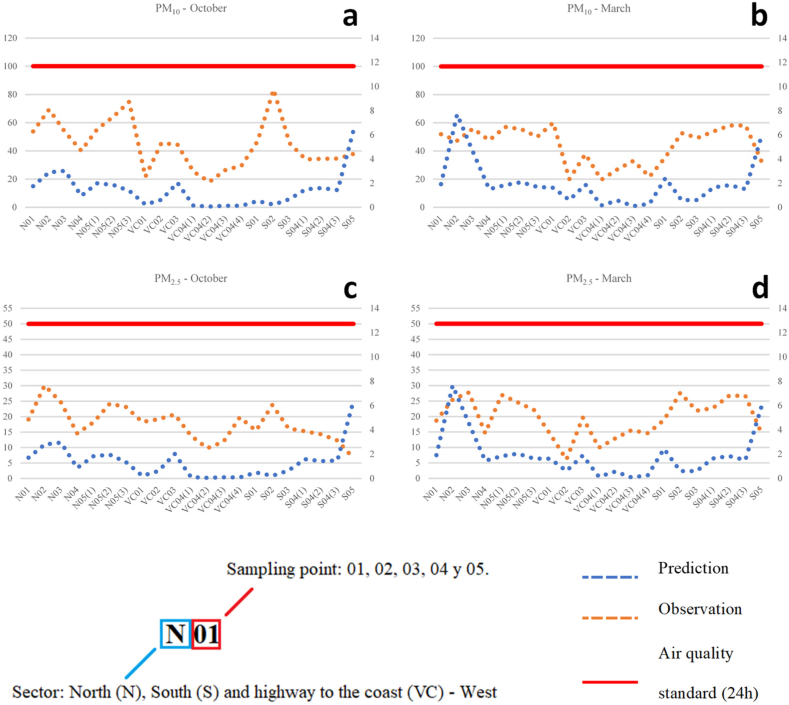
Daily average concentrations (μg/m^3^) of observation data and prediction results: PM_10_ October (a), PM_10_ March (b), PM_2.5_ October (c), PM_2.5_ March (d).

In a very general sense, it can be observed from Fig. 8 that, although there are considerable variations between the observed and simulated values in some of the sampling sites, the relative spatial variation was comparable in some of them**.** Meanwhile, better approximations were observed for PM_2.5_ (Fig. 8c and d) than for PM_10_ (Fig. 8a and b). This was reasonable because the dust sources not considered in this study from the mining and transportation sectors (non-exhaust emissions) had a greater impact on PM_10_ [70]. In both cases, the modeled values were substantially lower than the observed values. This disagreement might have related to the contribution of non-local sources, non-anthropogenic emissions, and secondary PM components that were not considered in this modeling exercise.

Nonetheless, the limitations of the simulation conducted were less important when focusing on analyzing the relative contribution of the different sources, as in this context, the absolute values of the deviations of the model were less influential for the interpretation of the results.

### 3.4 Analysis of source contribution

**Fig. 9:**
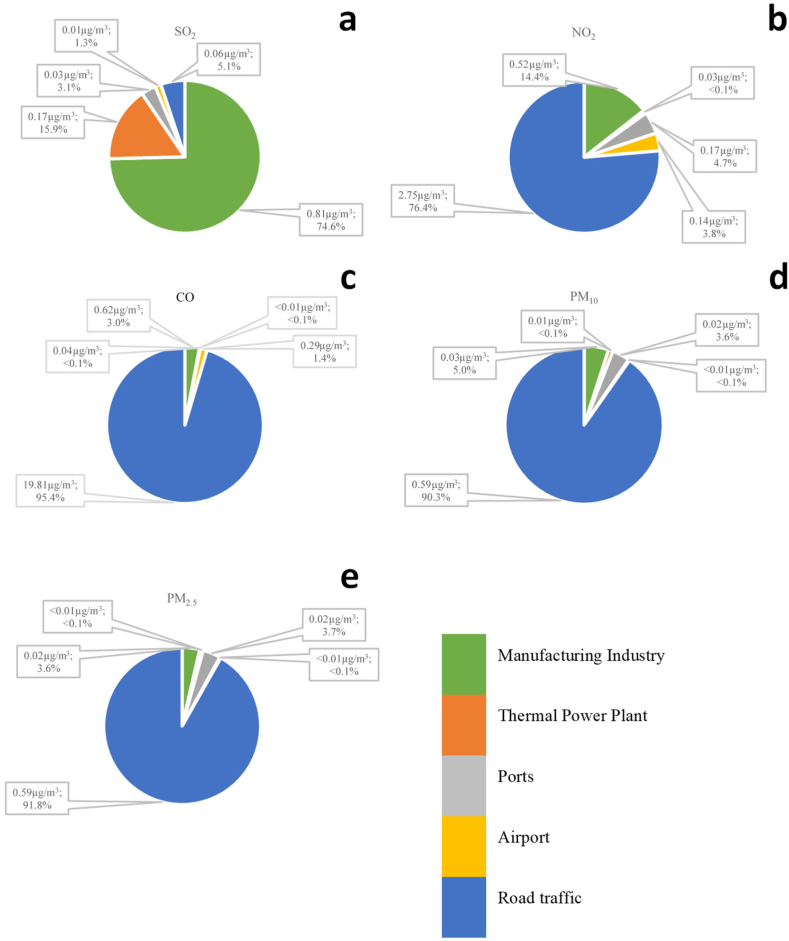
Sector contribution to the annual mean ambient concentration (μg/m^3^) averaged for the modeling domain for the main pollutants: SO_2_ (a), NO_2_ (b), CO (c), PM_10_ (d), and PM_2.5_ (e).

Fig. 9 shows the contribution of each source to the annual mean concentrations for the entire modeling area. This confirms that the city is strongly dominated by road traffic, except for the case of SO_2_ (Fig. 9a). For SO_2_, the main contribution was from the manufacturing sector at 75 % per year, followed by the thermal power plant sector at 16 %. The case of NO_2_, CO, PM_10_, and PM_2.5_, the contribution was mainly from the transport sector, with contributions of 76 %, 96 %, 90 %, and 92 %, respectively. In some areas of the city, these shares may be larger than those discussed in Section 3.1 regarding emissions. This result was consistent with those of other source apportionment studies conducted in urban areas [23].

**Fig. 10:**
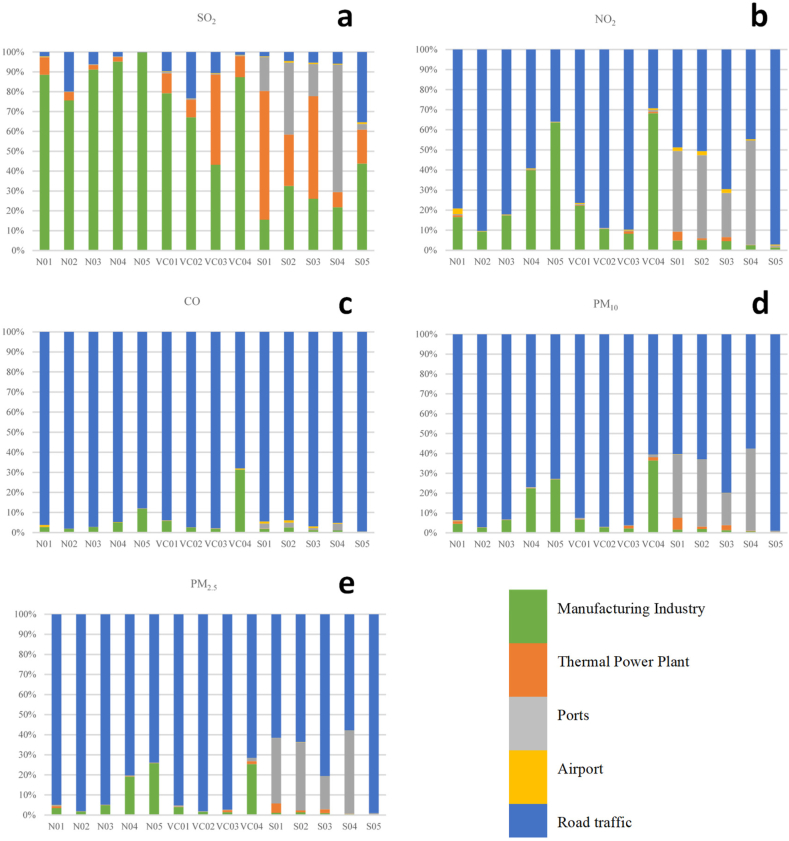
Percentage of annual average contribution from the air quality model at each monitoring station for the main pollutants: SO_2_ (a), NO_2_ (b), CO (c), PM_10_ (d), and PM_2.5_ (e).

Fig. 10 shows the contribution of the annual concentration by source type at each of the 14 monitoring stations defined in the measurement campaign. In the case of SO_2_ (Fig. 10a), the largest contribution came from the manufacturing sector, where the annual contribution ranges from 75 % to 99 % at the northern stations. This last value corresponded to the NO5 monitoring point located in the north, due to the component of the prevailing wind from the southwest and the relative location of a group of industries with significant emissions (southwest of this location). Regarding the thermoelectric plant sector, it should be noted that, at the monitoring points, SO1 reached a value of 64 % and 45 % at point VC03, which corresponded to the presence of thermoelectric plants with the highest generations in the city located southwest of these locations (Fig. 3).

In the case of the other pollutants, specifically NO_2_, CO, PM_10_, and PM_2.5_, the most significant contribution was from the transport sector, where the mean annual contribution was between 30 % and 97 % for the former, between 68 % and 99 % for the second one, between 60 % and 99 % for PM_10_, and PM_2.5_**.** The largest contributions were obtained from the center and northwest of the city. Because emissions from road traffic are released at the ground level, apportionment is less affected by wind patterns and is closely related to the spatial pattern of emissions [31].

The results summarized in Fig. 9, Fig. 10 indicated that the transport sector significantly dominated the ambient concentrations of all pollutants, except for SO_2_. Regarding particulate matter, the results presented here should be interpreted with caution. As mentioned in the methodology section, this study did not include measurements from the quarry sector, which is considered an important source of particulate matter emissions in the city, especially PM_10_. However, studies have shown that non-exhaust PM emissions from the transportation sector contribute significantly [52,71,72].

## 4 Limitations

The methodology developed in this study provides the first image of source apportionment in Guayaquil. However, these results should be interpreted within the context of methodological limitations. The most relevant results are summarized as follows.

• In addition to the output of the WRF forecast model, the evaluation of the CALMET diagnostic meteorological model should consider the input of information from surface meteorological stations (hybrid processing) to improve the performance of the meteorological and concentration fields.
• The spatial distribution of emissions from the transport sector should include detailed information on traffic fluxes according to the road type [73]. However, modeling this type of source requires an air quality model that considers mobile sources as line sources rather than area sources.
• Missing emission sources, including non-anthropogenic sources (vegetation, sea salt, etc.), mining, and non-exhaust emissions from road traffic, should be included to accurately describe the relative importance of the main sectors in Guayaquil. The influence of non-local sources, that is, those outside the innermost modeling domain, is also wholly neglected.
• The chemical transformation process was not considered in the simulation (except for the conversion of NO to NO_2_), with the consequent omission of the contribution of secondary pollutants to air quality in the city, which is a vital issue mainly for PM_2.5_ [55]. For the same reason, O_3_ was excluded from this study.
• The air quality modeling system could not be assessed because of a lack of measurements in the study area. Air quality data from further experimental campaigns or monitoring stations in a future municipal air quality network would allow a better understanding of the model performance. They may provide meaningful data for characterizing background pollution levels.

## 5 Conclusions and future research

In this study, a source distribution study was conducted in Guayaquil, Ecuador, by applying an air quality modeling system (WRF/CALMET/CALPUFF). To the best of our knowledge, this is the first study of this type in which the emissions of five relevant sectors within city limits have been considered: manufacturing, thermal power plants, ports, airports, and road transport.

Despite the methodological limitations mentioned in the previous section, road traffic was clearly identified as the most important emission source in the city, and our results suggest that policies aimed at reducing emissions from mobile sources may be particularly effective in meeting Ecuador's current ambient air quality standards. For instance, source apportionment analysis revealed that more than 90 % of PM_2.5_ and PM_10_ levels is related to that road traffic. Considering that 82 % of PM emissions within this sector come from public transport buses [31], the transition from a fuel-powered to an electric bus fleet may be the most effective measure for improving air quality in the city and reducing exposure. In the specific case of SO_2_, thermal power plants and manufacturing are the sectors with the greatest improvement potential. According to the inventory developed in this study, the substitution of fuel oils No. 4 and 6 would have a huge impact on curbing SO_2_ emissions and improving the ambient concentration of this pollutant in the city, as fuel oil No. 4 is a blend of distilled and residual oil, whereas fuel oil No. 6 is a residual crude oil known as Bunker, both containing significant amounts of sulfur. The emissions inventory revealed that the SO_2_ emissions generated by burning fuel oils No. 4 and No. 6 were particularly relevant in sectors such as power plants and manufacturing.

The analysis of the results at 14 specific locations along the modeling domain showed different relative weights for the contributions of the analyzed sectors. This information should be considered when designing a permanent air-quality monitoring network in Guayaquil that includes a balanced mix of stations. Nonetheless, social factors such as population distribution and environmental justice should be considered in addition to environmental criteria. A specific study on the design of such a monitoring system is considered as the main follow-up of this study. Further research should address the limitations of this study to obtain more reliable outcomes.

## Data availability statement

Data will be made available on request.

## CRediT authorship contribution statement

**Mario Patiño-Aroca:** Writing – review & editing, Writing – original draft, Visualization, Resources, Methodology, Conceptualization. **Tomás Hernández-Paredes:** Visualization, Methodology, Investigation. **Carlos Panchana-López:** Visualization, Methodology, Investigation. **Rafael Borge:** Writing – review & editing, Writing – original draft, Supervision, Project administration, Conceptualization.

## Declaration of competing interest

The authors declare that they have no known competing financial interests or personal relationships that could have appeared to influence the work reported in this paper.
