# Supplementary material for: Source apportionment of ambient pollution levels in Guayaquil, Ecuador
Source: Heliyon. 2024 May 21;10(11):e31613. doi: 10.1016/j.heliyon.2024.e31613 (PMC11154214; doi:10.1016/j.heliyon.2024.e31613)
Supplement: Multimedia component 1 [file mmc1.pdf]

SUPPLEMENTARY MATERIAL

Source apportionment of ambient pollution levels in Guayaquil, Ecuador

Mario Patiño-Aroca [a, b], Tomás Hernández-Paredes [a, c], Carlos Panchana-López [a], Rafael Borge [b]*

*[a] Escuela Superior Politécnica del Litoral, **ESPOL**, Campus Gustavo Galindo, Km 30.5 Vía Perimetral, Guayaquil, 090902, Ecuador.*

[b] Department of Chemical & Environmental Engineering, Universidad Politécnica de Madrid (UPM), C/ José Gutiérrez Abascal 2, 28006 Madrid, Spain.

[c] Universidad Agraria del Ecuador, Facultad de Ciencias Agrarias "Dr. Jacobo Bucaram Ortiz", Av. 25 de Julio y Pío Jaramillo, P.O. Box 09-04-100, Guayaquil, Ecuador.

**Corresponding author:* rafael.borge@upm.es

Table S1. Manufacturing industries of Guayaquil year 2018

ISIC Rev. 4 with two digits	Description ISIC Rev. 4 with two digits		Number of industries
C10	Manufacture of food products		53
C11	Manufacture of beverages		7
C13	Manufacture of textiles		2
C14	Manufacture of wearing apparel		4
C17	Manufacture of paper and paper products		12
C18	Printing and reproduction of recorded media		7
C19	Manufacture of coke and refined petroleum products		4
C20	Manufacture of chemicals and chemical products		20
C21	Manufacture of basic pharmaceutical products and pharmaceutical preparations		10
C22	Manufacture of rubber and plastics products		25
C23	Manufacture of other non-metallic mineral products.		9
C24	Manufacture of basic metals		6
C25	Manufacture of fabricated metal products, except machinery and equipment		13
C27	Manufacture of electrical equipment		11
C28	Manufacture of machinery and equipment n.e.c.		3
C30	Manufacture of other transport equipment		4
C31	Manufacture of furniture		1
C32	Other manufacturing		2
C33	Repair and installation of machinery and equipment		3
		Total	196

Note: ISIC=International Standard Industrial Classification.

Manufacturing emission inventory

The emission inventory for manufacturing industry was obtained considering the method of emission factors through equation 1:

$$E = FE * A \quad (1)$$

Where E is emissions, A is the activity rate and FE the emission factor.

The Tables S2, S3 and S4 show the emission factors for calculating emissions by process and combustion, and Table A5 shows the properties of the fuels. The emissions factors were obtained of the Part B: Sectoral Guidance Chapters del EMEP/EEA Air pollutant emission inventory guidebook 2019, section: 1. Energy, subsection: 1.A combustion, sub-chapter: 1.A.2 manufacturing industries and construction (combustion) and 1.A.4 small combustion; section: Industrial processes and product use, subsection: 2.A mineral products, 2.B chemical industry and 2.C metal production.

For the SO_2 combustion emissions, was obtained considering the method of mass balance, assuming complete oxidation of sulfur to sulfur dioxide as shown in equation 2.

$$E=C_{fuel}*S*\frac{MW_{SO2}}{MW_S} \quad (2)$$

Where E is the SO_2 emission rate, C_{fuel} is the fuel consumption rate, S is the sulfur content by mass in the fuel, and MW_{SO2} and MW_S are the molecular weights of sulfur dioxide and sulfur, respectively.

Table S2. Emissions factors used in the manufacturing process emissions inventory.

NFR		Table	Unit	SO_2	NO_x	CO	PM_{10}	$PM_{2.5}$
2.A.1	Cement production	3-1	g/ton clinker	-	-	-	234*	130*
2.A.2	Lime production	3-1	g/ton lime	-	-	-	3500	700
2.A.3	Glass production	3-1	g/ton glass	-	-	-	270	240
2.B	Chemical industry	3-47	g/ton produced	-	-	-	24**	18**
		3-29	kg/ton Urea	-	-	-	1.2	0.9
2.C.1	Iron and steel production	3-15	g/ton steel	60	130	1700	24	21
2.C.3	Aluminium production	3-4	kg/ton aluminium	-	-	-	1.4	0.55
2.C.6	Zinc production	3-4	g/ton zinc	-	-	-	340	255
2.H.2	Food and beverages industry	3-10	g/ton grains	-	-	-	24	-

Note: NFR= Nomenclature For Reporting.

* Considering a particle control system of n =80% (Table 3-2).

**PM_{10} and $PM_{2.5}$ assumed to be 0.8xTSP and 0.6xTSP.

Source: European Environment Agency [EEA], 2019.

Table S3. Emissions factors (g/GJ) used in the manufacturing combustion emissions inventory.

Boiler	Fuel	NFR	Table	NO_x	CO	PM_{10}	$PM_{2.5}$
≤ 50 KWth	Fuel Oíl #2	1.A.4	3-18	69.0	3.7	1.5	1.5
≤ 1MWth	Fuel Oíl #4	1.A.4	3-24	100.0	40.0	3.0	3.0
> 1MWth	Fuel Oíl #6	1.A.4	3-25	100.0	40.0	40.0	30.0
>50 kWth to <=1 MWth	Gas Natural	1.A.4	3-26	73.0	24.0	0.5	0.5
≤ 50 KWth	Petroleum Liquid Gas	1.A.2	3-3	74.0	29.0	0.8	0.8
<1 MWth	Petroleum Coke	1.A.4	3-23	165.0	350.0	78.0	70.0

Note: NFR= Nomenclature For Reporting.
Source: European Environment Agency [EEA], 2019.

Table S4. Emission factors used in industries with a furnace as an external combustion source.

SNAP	NFR	Table	Unit	SO_2	NO_x	CO	PM_{10}	$PM_{2.5}$
Combustion in manufacturing industries	1.A.2.f.i	3-24	g/ton clinker	374	1241	1455	-	-
		3-26	g/ton glass	1960	2930	6.13	-	-

Note: NFR= Nomenclature For Reporting.
Source: European Environment Agency [EEA], 2019.

Table S5. Properties of fuels used in the inventory of manufacturing and thermoelectric generation plants.

Properties	Unit	Fuel Oíl #2	Fuel Oíl #4	Fuel Oíl #6	Gas Natural	Petroleum Liquid Gas	Petroleum Coke
ash content	%	0.010	0.100	0.038	-	-	6.760
sulfur content	%	0.230	1.490	1.550	0.002	0.000	0.520
density	g/cm^3	0.852	0.961	0.961	0.633	0.528	-
LCV	MJ/kg	45.426	40.798	43.324	-	45.269	32.000

Notes: NFR= Nomenclature For Reporting.
LCV= Lower calorific value.
Sources: Corporación Eléctrica del Ecuador [CELEC EP], 2018.
EP PETROECUADOR, 2018.

Thermal power plant emission inventory

The emission inventory for the thermal power plant was obtained also considering the method of emission factors through equation 1. In the same way, the properties of the fuels shown in Table S5.

In the Table S6 shows the thermal power plant with their respective emission sources. The emissions factors show in the Table S7, were obtained of the Part B: Sectoral Guidance Chapters del EMEP/EEA Air pollutant emission inventory guidebook 2019, section: 1. Energy, subsection: 1.A combustion, sub-chapter: 1.A.1 energy industries.

Table S6. Sources of emissions from the thermal power plant.

Thermal Power Plant	Thermoelectric Plant type	No. sources	Source Code
Aníbal Santos	Turbosteam	6	V1-CAS
	Turbogas		G6-CAS
	Turbogas		G5-CAS
	Turbogas		G3-CAS
	Turbogas		G2-CAS
	Turbogas		G1-CAS
Álvaro Tinajero	Turbogas	1	G1-CAT
Trinitaria	Turbosteam	1	TV1
Gonzalo Zevallos	Turbosteam	3	TV-3
	Turbosteam		TV-2
	Turbogas		TG-4
Enrique García	Turbogas	1	TG-5
Generoca	Internal Combustion Engine	8	U8
	Internal Combustion Engine		U7
	Internal Combustion Engine		U6
	Internal Combustion Engine		U5
	Internal Combustion Engine		U4
	Internal Combustion Engine		U3
	Internal Combustion Engine		U2
	Internal Combustion Engine		U1
Total Emission Sources		20	

Sources: CELEC EP, 2018.
Agencia de Regulación y Control de Electricidad [ARCONEL], 2018.

Table S7. Emissions factors (g/GJ) used in the thermoelectric generation inventory.

Description	Fuel	NFR	Table	NO_x	CO	PM_{10}	$PM_{2.5}$
Dry bottom boilers	Fuel Oíl #4 y #6	1.A.1	3-11	142	15.1	25.2	19.3
Gas turbine	Fuel Oíl #2	1.A.1	3-18	398	1.49	1.95	1.95
ICE	Fuel Oíl #2	1.A.1	3-19	942	130	22.4	21.7

Notes: NFR= Nomenclature For Reporting.
ICE= Internal Combustion Engine.
Source: European Environment Agency [EEA], 2019.

Airport and ports emission inventory

The emission inventory for Airport activity was obtained considering the steps of Landing Take Off (LTO), Taxi in and Taxi Out, with the exception of Climb Cruise Descent (CCD) step, because of this it is performed mayorly out of domain area of study, above mixture height.

The amount of LTO cycles performed, added to with time suggested for the steps of Taxi-In/Taxi-Out, reported by airports management office, was used for inventory construction, implementing Part B: Sectoral Guidance Chapters del EMEP/EEA Air pollutant emission inventory guidebook 2019, section: 1.A combustion, sub-chapter: 1.A.3.a Aviation, Annex 5 - LTO emissions calculator 2019_2020. Finally, results obtained for each aircraft are aggregated to obtain the total emissions inventory for pollutants mentioned as follow SO_2, NO_x, CO, PM_{10} y $PM_{2.5}$.

Despite of there was not available all the data information required to apply the detailed EMEP/EEA methodology for the shipping emissions, the information collected from public and private sources was completed with data assumptions based on bibliography and with the aid of regression equations proposed by Carlos Trozzi to worldwide ships fleet (Trozzi, 2010), grouped by ship category: Liquid Bulk Ships, Dry Bulk Carriers, Containers, General Cargo, Ro Ro Cargo, Passenger, Fishing, Others. Finally, the results obtained for each ship category was aggregated to obtain the total emission inventory for pollutants SO_2, NO_x, CO, PM_{10} y $PM_{2.5}$ related to port activity and its traffic intensity. Table S8 show the emission factors considered to emission inventory construction for ports.

Table S8. Emissions factors (g/kWh) used in marine emissions inventory.

Engine	Phase	Engine Type	Fuel Type	NFR	Table	SO_2	NO_x	CO	PM_{10}	$PM_{2.5}$
Main	Manoeuvring / Hotelling	High-speed diesel	Bunker Fuel Oil	1.A.3.d	3-1 / 3-2 / 3-10	7	9.5	1.7	2.4	2.4
			Marine Gas Oil	1.A.3.d	3-1 / 3-2 / 3-10	0.9	8.9	1.7	0.9	0.9
		Medium-speed diesel	Bunker Fuel Oil	1.A.3.d	3-1 / 3-2 / 3-10	7	10.4	1.7	2.4	2.4
			Marine Gas Oil	1.A.3.d	3-1 / 3-2 / 3-10	0.9	9.9	1.7	0.9	0.9
		Slow-speed diesel	Bunker Fuel Oil	1.A.3.d	3-1 / 3-2 / 3-10	6.5	13.5	1.6	2.4	2.4
			Marine Gas Oil	1.A.3.d	3-1 / 3-2 / 3-10	0.8	12.7	1.5	0.9	0.9
Auxiliary	Manoeuvring / Hotelling	High-speed diesel	Bunker Fuel Oil	1.A.3.d	3-1 / 3-2 / 3-10	6.8	10.8	1.7	0.8	0.8
			Marine Gas Oil	1.A.3.d	3-1 / 3-2 / 3-10	0.9	10.2	1.6	0.3	0.3
		Medium-speed diesel	Bunker Fuel Oil	1.A.3.d	3-1 / 3-2 / 3-10	6.8	13.7	1.7	0.8	0.8
			Marine Gas Oil	1.A.3.d	3-1 / 3-2 / 3-10	0.9	13	1.6	0.3	0.3

Note: NFR= Nomenclature For Reporting.
Source: European Environment Agency [EEA], 2019.

Table S9. Activity data used for the temporal allocation of emissions from LTO and shipping.

Month	Summary of Activity 2018	
	Air Transportation [Flight]	Marine Transportation [Arrival]
January	5684	144
February	4779	141
March	5950	148
April	6143	154
May	6213	147
June	6168	147
July	6425	146
August	6622	145
September	6227	148
October	6595	159
November	6343	153
December	6350	164
Total	73499	1796

On-road emission (from Patiño-Aroca et al., 2022)

In this study, the emissions inventory was obtained from an experimental field campaign ("Guayaquil Vehicle Activity Study") to obtain information on the dynamic composition and technological distribution of the fleet and the driving and start/stop patterns of the engine, whose results were fed to the IVE emissions model.

The study considered five categories of vehicles: private passenger (PC), taxis, motorcycles (2w), buses, and trucks. The contribution to the total emission by vehicle category is shown in Table S10.

In this study, the total emissions were distributed with a spatial resolution of 1 km x 1 km and a temporal resolution of 1 h, for which a top-down methodology was used to estimate a spatial distribution of their emissions.

Table S10. Estimated emissions contribution by vehicle category, in 10^3 t/year (Patiño-Aroca et al, 2022).

Category	% of vehicles	% VKTs	CO	NO_x	PM_{10}	$PM_{2.5}$	SO_2
PV	68.6%	52.0%	136.00	12.56	0.42	0.42	0.33
Taxi	8.8%	22.0%	82.93	4.78	0.019	0.019	0.11
Motorcycle	10.1%	7.3%	11.95	0.67	0.13	0.13	0.01
Truck	5.0%	8.1%	1.22	5.67	0.81	0.81	0.05
Bus	7.5%	10.6%	5.02	22.7	6.28	6.28	0.19
Total	100%	100%	237.12	46.38	7.66	7.66	0.69

Table S11. WRF meteorological data for CALPUFF CALMET-Ready WRF data Jan 08, 2020

**Met Data Type:**	CALMET-Ready WRF Data (3D.DAT Format)
**Order Start-End Date:**	Jan 01, 2015 to Dec 31, 2018
**Center Point:**	Latitude: 2.15 S - Longitude: 79.88 W
**Datum:**	WGS 84
**UTM Zone:**	-17
**WRF Resolution:**	4 km
**WRF Domain Size:**	50 x 50 km
**WRF Vertical Levels:**	35 (lowest level at ~20 m above ground level)
**Site Time Zone:**	UTC-0500
**Location:**	Guayaquil, Ecuador

Table S12. WRF nested domain grids – 12km & 4km, 50x50km domain.

Domain	Resolution (km)	Number of Grid Points in X and Y
Domain 1	108	31 x 31
Domain 2	36	31 x 31
Domain 3	12	31 x 31
Domain 4	4	31 x 31

Table S13. Physics options used for WRF modeling.

#	Type	Options Used
1	Microphysics	WRF Single-moment 3-class scheme mp_physics = 3
2	Long-wave Radiation	RRTMG Longwave scheme ra_lw_physics = 4
3	Short-wave Radiation	RRTMG Shortwave scheme ra_sw_physics = 4
4	Surface Layer	Revised MM5 scheme sf_sfclay_physics = 1
5	Land Surface	Unified Noah Land Surface model sf_surface_physics = 2
6	Planetary Boundary Layer	Yonsei University (YSU) scheme bl_pbl_physics = 1
7	Cumulus parameterization	Kain-Fritsch (grid size > 10km only) cu_physics = 1

Table S14. Manufacturing industries with significant sources (combustion and process).

ISIC Rev. 4 with two digits	Description ISIC Rev. 4 with two digits
C10	Manufacture of food products
C11	Manufacture of beverages
C17	Manufacture of paper and paper products
C22	Manufacture of rubber and plastics products
C23	Manufacture of other non-metallic mineral products
C24	Manufacture of basic metals

Table S15. Physical and operational parameters and emission rates for manufacturing modeling (combustion and process emissions).

ISIC Rev.4 with two digits	Industries	No. of emission sources by industry	Stack Data		Gas Data		SO_2	NO_x	CO	PM_{10}	$PM_{2.5}$
			Height [m]	Diam [m]	Exit_Vel [m/s]	Exit_Temp [K]	[g/s]	[g/s]	[g/s]	[g/s]	[g/s]
C10	No. 1	Boiler 1	11.50	0.77	9.42	485.60	3.45	0.47	0.19	0.02	0.02
		Boiler 2	10.47	0.63	8.84	469.00	3.45	0.47	0.19	0.02	0.02
	No. 2	Boiler 1	4.00	0.45	3.80	502.95	3.29	0.45	0.18	0.01	0.01
		Boiler 2	5.00	0.55	5.03	496.85	3.29	0.45	0.18	0.01	0.01
		Boiler 3	4.00	0.45	3.84	508.25	3.29	0.45	0.18	0.01	0.01
		Boiler 4	6.00	0.60	7.19	507.35	3.29	0.45	0.18	0.01	0.01
C11	No. 3	Boiler 1	9.00	0.60	8.84	454.25	12.07	1.65	0.66	0.05	0.05
	No. 4	Boiler 1	11.10	0.38	9.42	443.25	2.45	0.33	0.13	0.01	0.01
C17	No. 5	Boiler 1	12.00	0.48	8.84	449.25	3.88	0.53	0.21	0.02	0.02
C22	No. 6	Boiler 1	8.30	0.50	7.43	500.55	3.71	0.51	0.2	0.02	0.02
C23	No. 7	Kiln 1	45.00	0.98	19.40	639.15	10.70	15.99	0.03	1.47	1.31
	No. 8	Kiln 1	99.00	3.00	20.10	423.30	11.62	38.57	45.22	1.45	0.81
		Kiln 2	129.00	3.70	22.40	362.30	11.62	38.57	45.22	1.45	0.81
C24	No. 9	Boiler 1	27.00	1.90	8.84	468.85	0.06	0.12	1.57	0.02	0.02
		Boiler 2	40.00	4.24	8.84	468.85	0.06	0.12	1.57	0.02	0.02

Sources: Muy Ilustre Municipalidad de Guayaquil, 2018.
U.S. Environmental Protection Agency [US EPA][1].

[1] https://www.cmascenter.org/smoke/documentation/4.5/html/ch08s09s03.html#tbl_input_pstk

Table S16. Physical and operational parameters and emission rates for thermal power plant modeling.

Source Code	Stack Data		Gas Data		SO_2 [g/s]	NO_x [g/s]	CO [g/s]	PM_{10} [g/s]	$PM_{2.5}$ [g/s]
	Height [m]	Diam [m]	Exit_Vel [m/s]	Exit_Temp [K]					
V1CAS	30.00	3.60	15.80	413.00	3.01E-05	1.18E-04	4.43E-07	5.80E-07	5.80E-07
G1CAS	5.70	2.50	20.00	569.11	3.93E-01	1.54E+00	5.78E-03	7.57E-03	7.57E-03
G2CAS	5.70	2.50	20.00	569.11	1.94E-03	7.64E-03	2.86E-05	3.74E-05	3.74E-05
G3CAS	5.70	2.50	20.00	569.11	1.36E-01	5.34E-01	2.00E-03	2.62E-03	2.62E-03
G5CAS	4.70	4.80	20.95	649.43	9.89E-02	3.89E-01	1.46E-03	1.90E-03	1.90E-03
G6CAS	15.00	3.93	19.51	711.80	2.46E-01	9.69E-01	3.63E-03	4.75E-03	4.75E-03
G1CAT	18.40	2.38	50.00	722.85	1.75E+00	6.88E+00	2.58E-02	3.37E-02	3.37E-02
TV1	60.00	4.65	16.60	396.20	1.17E+02	2.27E+01	2.41E+00	4.02E+00	3.08E+00
TV2	30.00	3.60	25.50	439.10	6.86E+01	1.34E+01	1.42E+00	2.37E+00	1.81E+00
TV3	30.00	3.60	25.50	439.10	9.64E+01	1.88E+01	1.99E+00	3.32E+00	2.55E+00
TG4	8.00	2.57	20.00	693.00	3.65E-01	1.43E+00	5.37E-03	7.02E-03	7.02E-03
TG5	15.84	6.12	21.10	631.00	1.64E+00	6.44E+00	2.41E-02	3.15E-02	3.15E-02
GEN_MCI_2	25.00	2.30	18.04	568.15	4.86E+00	1.12E+00	1.25E-01	1.65E-01	1.28E-01
GEN_MCI_1	25.00	2.30	18.04	568.15	4.13E+00	9.15E-01	1.02E-01	1.40E-01	1.08E-01

Table S17. Physical and operational parameters and emission rates for marine ports

Source Code	Stack Data		Gas Data		SO_2	NO_x	CO	PM_{10}	$PM_{2.5}$
	Height [m]	Diam [m]	Exit_Vel [m/s]	Exit_Temp [K]	[g/s]	[g/s]	[g/s]	[g/s]	[g/s]
Port 1	22	1.2	25	558	7.36E-01	5.00E+00	7.98E-01	5.19E-01	5.19E-01
Port 2	22	1.2	25	558	9.58E-01	6.51E+00	1.04E+00	6.75E-01	6.75E-01
Port 3	22	1.2	25	558	5.46E-01	3.72E+00	5.93E-01	3.85E-01	3.85E-01
Port 4	22	1.2	25	558	1.77E+00	1.12E+01	1.78E+00	1.19E+00	1.19E+00
Port 5	22	1.2	25	558	2.82E-01	2.01E+00	3.22E-01	2.05E-01	2.05E-01

Table S18. Physical and operational parameters and emission rates for airport

Source Code	Area Source	Source Data				SO_2	NO_x	CO	PM_{10}	$PM_{2.5}$
		Height [m]	Length X [m]	Length Y [m]	Rotation Angle [deg]	$\left[\frac{g/s}{m^2}\right]$	$\left[\frac{g/s}{m^2}\right]$	$\left[\frac{g/s}{m^2}\right]$	$\left[\frac{g/s}{m^2}\right]$	$\left[\frac{g/s}{m^2}\right]$
AEROPTO4_A	1	0	20	200	30	7.60E-06	1.02E-04	1.62E-04	7.80E-07	7.80E-07
AEROPTO4_B	2	0	20	200	30	7.60E-06	1.02E-04	1.62E-04	7.80E-07	7.80E-07
AEROPTO4_C	3	0	20	200	30	7.60E-06	1.02E-04	1.62E-04	7.80E-07	7.80E-07
AEROPTO4_D	4	0	20	200	30	7.60E-06	1.02E-04	1.62E-04	7.80E-07	7.80E-07
AEROPTO4_E	5	0	20	200	30	7.60E-06	1.02E-04	1.62E-04	7.80E-07	7.80E-07
AEROPTO4_F	6	0	20	200	30	7.60E-06	1.02E-04	1.62E-04	7.80E-07	7.80E-07
AEROPTO4_G	7	0	20	200	30	7.60E-06	1.02E-04	1.62E-04	7.80E-07	7.80E-07
AEROPTO4_H	8	0	20	200	30	7.60E-06	1.02E-04	1.62E-04	7.80E-07	7.80E-07
AEROPTO4_I	9	0	20	200	30	7.60E-06	1.02E-04	1.62E-04	7.80E-07	7.80E-07
AEROPTO4_J	10	0	20	200	30	7.60E-06	1.02E-04	1.62E-04	7.80E-07	7.80E-07
AEROPTO4_K	11	20	20	200	30	7.60E-06	1.02E-04	1.62E-04	7.80E-07	7.80E-07
AEROPTO4_L	12	40	20	200	30	7.60E-06	1.02E-04	1.62E-04	7.80E-07	7.80E-07
AEROPTO4_M	13	60	20	200	30	7.60E-06	1.02E-04	1.62E-04	7.80E-07	7.80E-07
AEROPTO4_N	14	80	20	200	30	7.60E-06	1.02E-04	1.62E-04	7.80E-07	7.80E-07
AEROPTO4_O	15	100	20	200	30	7.60E-06	1.02E-04	1.62E-04	7.80E-07	7.80E-07
AEROPTO4_P	16	120	20	200	30	7.60E-06	1.02E-04	1.62E-04	7.80E-07	7.80E-07
AEROPTO4_Q	17	140	20	200	30	7.60E-06	1.02E-04	1.62E-04	7.80E-07	7.80E-07
AEROPTO4_R	18	160	20	200	30	7.60E-06	1.02E-04	1.62E-04	7.80E-07	7.80E-07
AEROPTO4_S	19	180	20	200	30	7.60E-06	1.02E-04	1.62E-04	7.80E-07	7.80E-07
AEROPTO4_T	20	200	20	200	30	7.60E-06	1.02E-04	1.62E-04	7.80E-07	7.80E-07
AEROPTO4_U	21	220	20	200	30	7.60E-06	1.02E-04	1.62E-04	7.80E-07	7.80E-07
AEROPTO4_V	22	240	20	200	30	7.60E-06	1.02E-04	1.62E-04	7.80E-07	7.80E-07
AEROPTO4_W	23	260	20	200	30	7.60E-06	1.02E-04	1.62E-04	7.80E-07	7.80E-07
AEROPTO4_X	24	280	20	200	30	7.60E-06	1.02E-04	1.62E-04	7.80E-07	7.80E-07
AEROPTO4_Y	25	300	20	200	30	7.60E-06	1.02E-04	1.62E-04	7.80E-07	7.80E-07

Table S19. Codes for receptors

Code	Coordinate X	Coordinate Y	Location description
N01	623243	9764592	Mercado de Sauces 9
N02	615779	9767152	Flor de Bastión UPC
N03	617681	9768264	UPC3 - Sector Montebello
N04	619314	9771529	Centro de salud - Pascuales
N05	616630	9772378	Ingreso a Tecnova
VC01	613232	9757742	Urb. Belo Horizonte
VC02	608446	9757742	Puerto Hondo
VC03	617910	9759798	Planta Nestlé - Av. Del Bombero
VC04	615001	9762942	ESPOL - Parqueadero FIMCP
S01	622274	9751860	Av. Padre Cayetano Tarruel y Los Esteros
S02	625573	9749832	Novacero
S03	623388	9753105	El Universo
S04	619438	9751729	TPG 1 - UPC
S05	621509	9755785	Sector iglesia Espíritu Santo

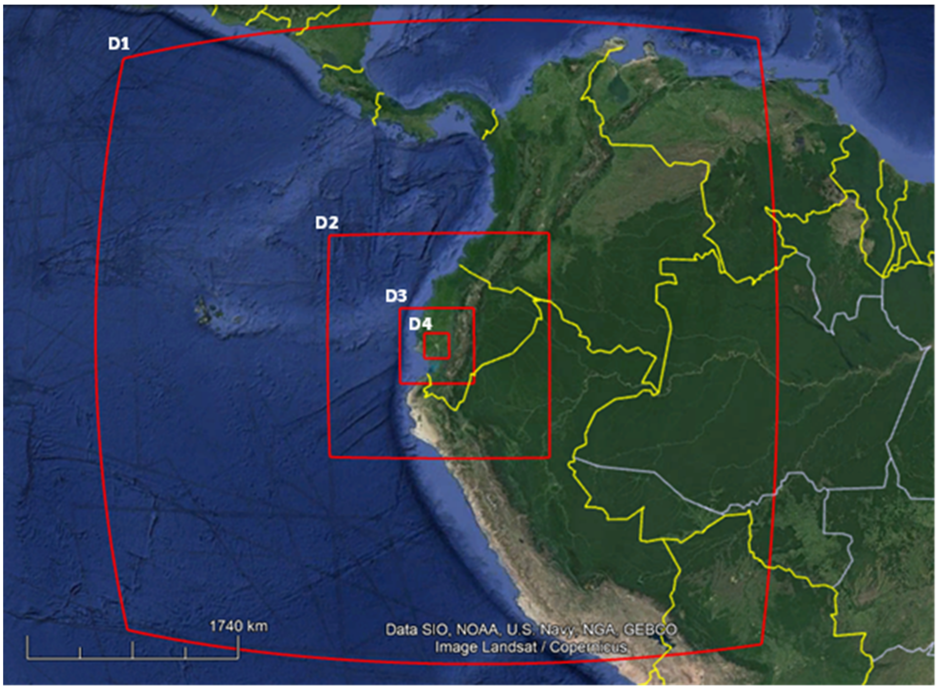

Figure S1. WRF modeling domains.

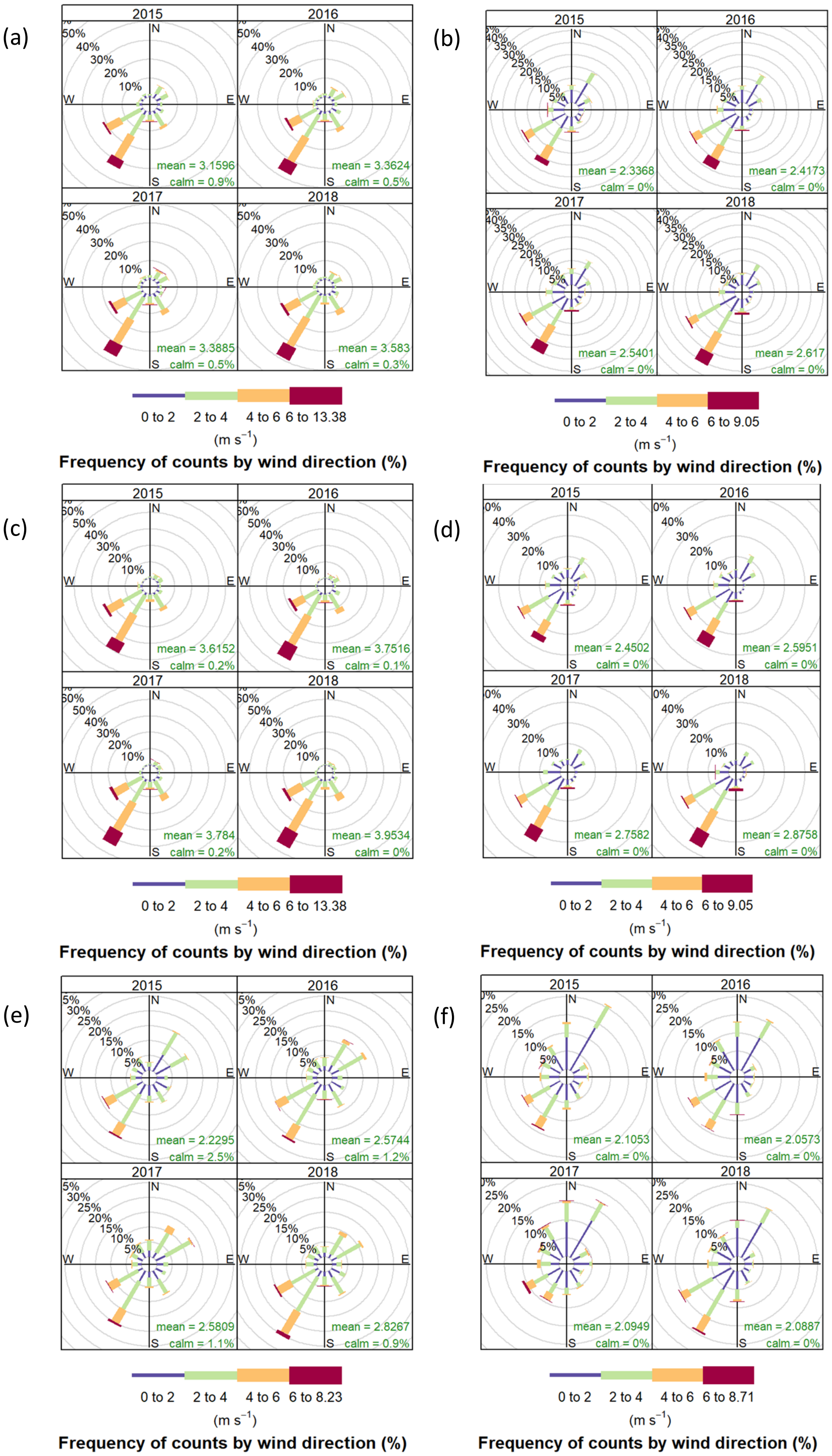

Figure S2. Observed (left) and modeled by WRF/CALMET (right) wind rose: annual (a) (b); dry season per year (c) (d); rainy season per year (e) (f).

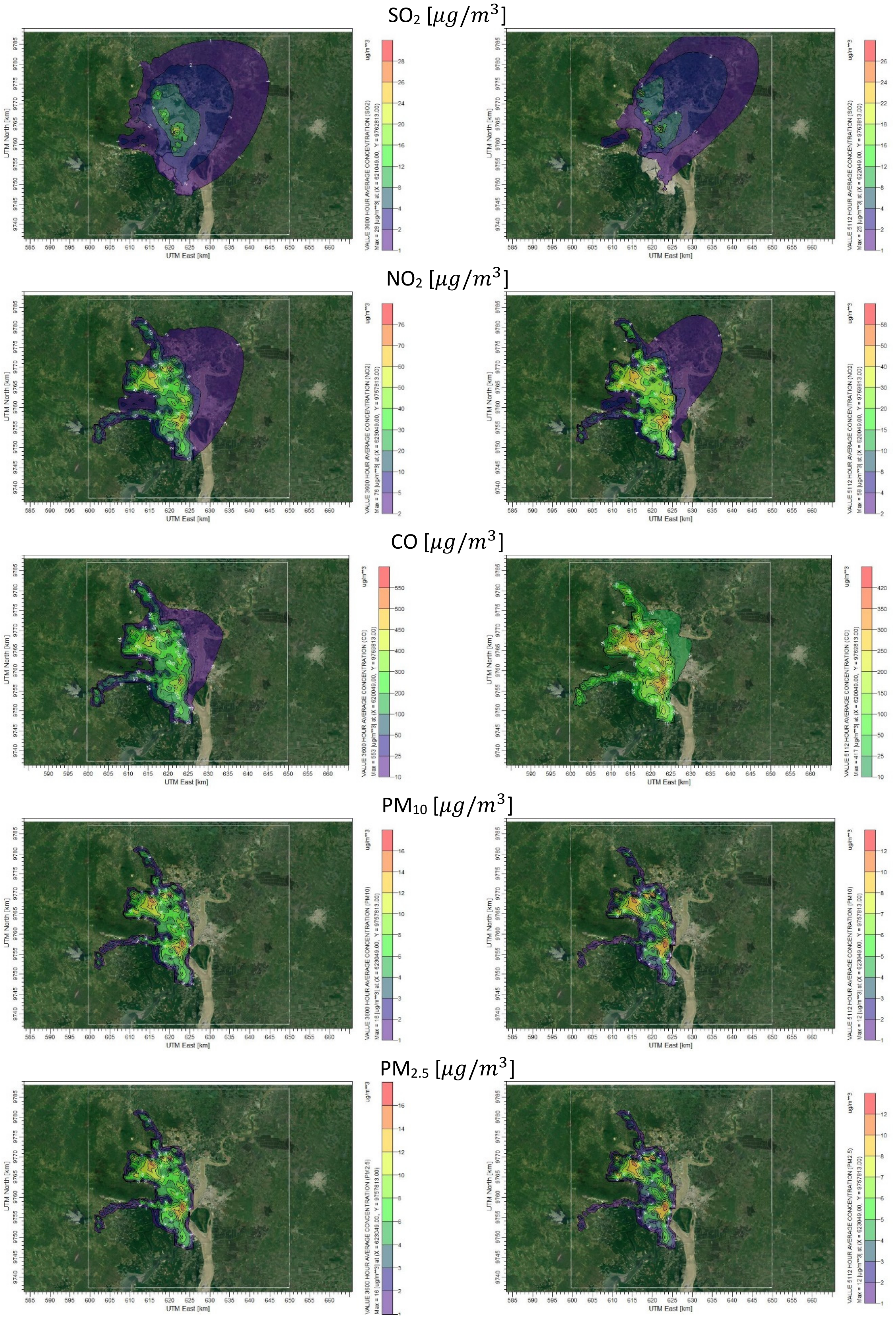

Figure S3. The spatial distribution of the simulated concentration fields. Rainy season (left) and dry season (right).

Statistics Indices:

$$MB = \frac{1}{N}\sum_{i=1}^{N}(M_i - O_i) \tag{1}$$

$$MAE = \frac{1}{N}\sum_{i=1}^{N}|M_i - O_i| \tag{2}$$

$$RMSE = \sqrt{\left(\frac{\sum_{i=0}^{N}(M_i - O_i)^2}{N}\right)} \tag{3}$$

$$IOA = 1 - \frac{N \times RMSE^2}{\sum_{i=1}^{N}(|M_i - \bar{O}| + |O_i - \bar{O}|)^2} \tag{4}$$

where:

M_i = modeled value

O_i = observed value

$\bar{O}$ = mean of observed value

N = number of paired values
